# ET-CORM Mediated Vasorelaxation of Small Mesenteric Arteries: Involvement of Kv7 Potassium Channels

**DOI:** 10.3389/fphar.2021.702392

**Published:** 2021-09-06

**Authors:** Danfeng Zhang, Bernhard M. Krause, Hans-Günther Schmalz, Paulus Wohlfart, Benito A. Yard, Rudolf Schubert

**Affiliations:** ^1^ Department of Nephrology, Endocrinology and Rheumatology, Fifth Medical Department of Medicine, Medical Faculty Mannheim, University of Heidelberg, Mannheim, Germany; ^2^ Department of Nephrology, the Second Hospital of Anhui Medical University, Hefei, China; ^3^ Department of Chemistry, University of Cologne, Cologne, Germany; ^4^ Diabetes Research, Sanofi Aventis Deutschland GmbH, Frankfurt, Germany; ^5^ European Center of Angioscience (ECAS), Research Division Cardiovascular Physiology, Medical Faculty Mannheim, Heidelberg University, Frankfurt, Germany; ^6^ Physiology, Institute of Theoretical Medicine, Medical Faculty, University of Augsburg, Augsburg, Germany

**Keywords:** vasorelaxation, carbon monoxide, potassium channels, rat, mesenteric arteries

## Abstract

Although the vasoactive properties of carbon monoxide (CO) have been extensively studied, the mechanism by which CO mediates vasodilation is not completely understood. Through-out published studies on CO mediated vasodilation there is inconsistency on the type of K^+^-channels that are activated by CO releasing molecules (CORMs). Since the vasorelaxation properties of enzyme triggered CORMs (ET-CORMs) have not been studied thus far, we first assessed if ET-CORMs can mediate vasodilation of small mesenteric arteries and subsequently addressed the role of soluble guanylate cyclase (sGC) and that of K-channels herein. To this end, 3 different types of ET-CORMs that either contain acetate (*rac*-**1** and *rac*-**4**) or pivalate (*rac*-**8**) as ester functionality, were tested *ex vivo* on methoxamine pre-contracted small rat mesenteric arteries in a myograph setting. Pre-contracted mesenteric arteries strongly dilated upon treatment with both types of acetate containing ET-CORMs (*rac*-**1** and *rac*-**4**), while treatment with the pivalate containing ET-CORM (*rac*-**8**) resulted in no vasodilation. Pre-treatment of mesenteric arteries with the sGC inhibitor ODQ abolished *rac*-**4** mediated vasodilation, similar as for the known sGC activator SNP. Likewise, *rac*-**4** mediated vasodilation did not occur in KCL pretreated mesenteric arteries. Although mesenteric arteries abundantly expressed a variety of K^+^-channels only Kv7 channels were found to be of functional relevance for *rac*-**4** mediated vasodilation. In conclusion the current results identified Kv7 channels as the main channel by which *rac*-**4** mediates vasodilation. In keeping with the central role of Kv7 in the control of vascular tone and peripheral resistance these promising *ex-vivo* data warrant further *in vivo* studies, particularly in models of primary hypertension or cardiac diseases, to assess the potential use of ET-CORMs in these diseases.

## Introduction

Carbon monoxide (CO), endogenously produced by heme oxygenases (HO), is considered to be an important signaling molecule participating in a variety of cellular functions (Romanski et al., 2012a; Olas, 2014; Naito et al., 2016). The interest for using CO as a potential therapeutic agent has increased considerably in the past decade as a number of *in vitro* and *in vivo* studies have corroborated the beneficial effect of CO in a variety of disease models (Guo et al., 2004; Musameh et al., 2007; Ferrándiz et al., 2008). Clinical trials to assess the feasibility and safety of therapeutic CO inhalation have been performed in patients with chronic obstructive pulmonary disease (NCT00122694) (Bathoorn et al., 2007) and in patients with sepsis-induced acute respiratory distress syndrome (NCT02425579) (Fredenburgh et al., 2018; Mayr et al., 2005).

While in phase I clinical studies CO was applied in its gaseous form, in recent years a number of different CO releasing molecules (CORMs) have been synthesized and successfully tested in *in vitro* and *in vivo* inflammation models. CORMs release CO either spontaneously or require specific triggering mechanisms. In order to deliver CO directly into cells and to gain a better control over CO release, we have introduced so called enzyme-triggered CORMs (ET-CORMs) (Romanski et al., 2011; Romanski et al., 2012a; Romanski et al., 2013). ET-CORMs are acyloxy-butadiene–Fe(CO)_3_ complexes that are relatively stable under physiological conditions. Once intracellular, the ester functionality of ET-CORMs is cleaved by hydrolytic enzymes (e.g., esterases), resulting in the formation of labile dienol iron carbonyl complexes that subsequently disintegrate under oxidative conditions to release CO, ferric ions and the corresponding enones (Romanski et al., 2011). CO release can be influenced by the type and position of the ester substituent of the ET-CORM as well as by the mother compound from which it is derived (Romanski et al., 2013). This concept also allows the design of protease specific CO release, thus paving avenues for the implementation of cell specific CO delivery (Sitnikov et al., 2015).

The HO-1/CO system plays prominent biological roles in the vasculature. CO is an often under-appreciated signaling molecule, acting on a wide variety of signaling pathways that regulate vasodilation (Koçer et al., 2018), inflammation (Ryter and Choi, 2016) as well as angiogenesis (Choi et al., 2017). CO administration in its gaseous form appears to reduce the contractility of pre-contracted arteries which may prevent or mitigate the occurrence of vasoconstrictor mediated spasm of arterial grafts in coronary artery bypass surgery in a cGMP dependent manner (Wang et al., 1997a; Achouh et al., 2005). CO delivery *via* CORMs also has beneficial effects on the vasculature, e.g., blunting of placental ischemia induced hypertension (George et al., 2017) and improvement of cerebrovascular dysfunction caused by neonatal seizures (Liu et al., 2015). Yet, vessel relaxation mediated by gaseous CO or CORM-2 may differ in their mode of action as suggested by Decaluwé et al. (Decaluwé et al., 2012). They postulated that CO relaxes vessels through activation of sGC and/or calcium-activated K^+^-channels, while it seems that CORM-2 induced vasodilatation much more depends on voltage-dependent rather than calcium-activated K^+^-channels. Participation of cGMP, potassium channels and nitric oxide (NO) has been postulated in the vasodilatory properties of the water soluble CORM-2 and CORM-3 (Al-Owais et al., 2017), yet the involvement of NO has been questioned by others (Failli et al., 2012; Alshehri et al., 2013). Because CO can cause concomitantly activation and inhibition of NO synthase, it seems that CORMs display context-dependent effects as they can directly dilate blood vessels, but also block NO-induced vasorelaxation (Alshehri et al., 2013).

Even though the role of K^+^-channels in CO mediated vasorelaxation is widely accepted, there exists controversy on the class of K^+^-channels by which this occurs. Whether different CORMs activate different K^+^-channels or if differences in CO mediated vasorelaxation are due to differences in the expression level of K^+^-channels in different vessels remains to be addressed. Based on the current understanding of the vasoactive properties of CO and CORMs, this study underlies the hypothesis that ET-CORMs are able to cause vasodilation of pre-contracted resistance vessels in a sGC and K^+^-channel dependent manner. The hypothesis was tested by studying vascular responses of small rat mesenteric arteries *ex-vivo*.

## Materials and Methods

### Drugs

Acyloxydiene complexes (ET-CORM) *rac-* **1**, *rac*-**4** and *rac-* **8** were synthesized as previously described (Romanski et al., 2011). Stock solutions were prepared in dimethyl sulfoxide (DMSO) and stored at −20°C. The parent ligand of *rac-* **4**, 2-cyclohexenone was included to assess whether vascular activity was mediated by released CO or by the by-products of *rac-* **4** cleavage. Methoxamine, acetylcholine (both dissolved in H_2_O), XE991 and glibenclamide (both dissolved in DMSO) were obtained from Sigma. Iberiotoxin and DPO-1 were obtained from Tocris (Wiesbaden-Norden-stadt, Germany). Stromatoxin (dissolved in H_2_O) was obtained from Alomone Labs (Jerusalem, Israel). BaCl_2_ (dissolved in H_2_O) was obtained from Riedel-de-Haën (Seelze, Germany).

### Animals

Adult, 8- to 12-week-old, male Wistar rats were obtained from Janvier (France; RRID: RGD_13,508,588). The animals were provided with food and water ad libitum and housed in a room with a controlled temperature and a 12-h light-dark cycle in IVC cages. The use of laboratory animals and all procedures included in this study were in accordance with the NIH Guide for the care and Use of Laboratory Animals. Approval for the use of laboratory animals in this study was granted by a governmental committee on animal welfare (I-17/17).

### Vessel Preparation

Rats were sacrificed under CO_2_ narcosis by decapitation. The mesentery was immediately removed and transferred to cold (4°C) physiological salt solution (PSS) composed of (in mM): NaCl 145; KCl 4.5; CaCl_2_ 0.1; MgSO_4_ 1.0; NaH_2_PO_4_ 1.2; EDTA 0.025; HEPES 5.0 (pH = 7.4). Mesenteric arteries (∼150 μm diameter and 2 mm long) were dissected and cleaned off fat and connective tissue. Mesenteric artery rings were mounted in a wire myograph (model 610M, Danish Myo Technology, Denmark). The organ bath was filled with 5 ml PSS consisting of (in mM) NaCl 120; NaHCO_3_ 26; KCl 4.5; CaCl_2_ 1.6; MgSO_4_ 1.0; NaH_2_PO_4_ 1.2; d-glucose 5.5; EDTA 0.025; HEPES 5.0, was heated to 37°C, and continuously oxygenated with carbogen (95% O_2_/5% CO_2_) to maintain pH at 7.4. In some experiments, the endothelium of mesenteric artery rings was removed by scratching the lumen of the vessel gently with a rat whisker. The successful functional removal of the endothelium was confirmed by the absence of acetylcholine-induced vasodilation of 1 μmol/L methoxamine pre-contracted arteries. Each mesenteric artery segment underwent a normalization procedure before the experiment. In order to obtain optimal responses of the vessels, the segments were stretched stepwise to a diameter corresponding to 90% of the diameter the vessel would have at a transmural pressure of 100 mmHg (13,3kPa) using the Lab Chart DMT (Mulvany and Halpern, 1977) Normalization module. All data acquisition and analysis were performed using LabChart (AD Instruments, United States). Viability of the vessel preparations was determined with methoxamine at 10 μM to test smooth muscle cell function. All drugs were added to the bath solution. Arterial tension is expressed as a percentage of the steady-state tension (100%) generated by 10 μM methoxamine.

### Gene Expression Analysis

Mesenteric arteries were isolated as described above, snap-frozen in liquid nitrogen and stored at −80°C. Total RNA isolation was performed using the RNeasy RNA isolation kit according to the manufacturer’s instruction. Further purification of RNA was performed according to the protocol provided by the manufacturer. The quantity and quality of the isolated RNA were controlled using an RNA 6000 nano kit (Agilent, Waldbronn, Germany). Only samples with an integrity RIN value > 7.5 were used for further analysis. Reverse transcription was performed using a high capacity RNA-to-cDNA Kit (Applied Biosystems, Weiterstadt, Germany). Real-time PCR was performed on potassium ion channel genes in parallel using TaqMan microfluidic card technology in a Viia7 thermocycler (ThermoFisher, Darmstadt, Germany) with a maximum of 40 cycles. These cards contained wells for specific channel genes and five reference (housekeeping) genes. All TaqMan primers were tested before by dilution experiments and used only when amplification efficacies were close to 100%. Threshold quantification cycles (Cq values) were obtained for each gene delivered by the manufacturer’s Viia7 software and further analyzed using the ArrayStudio software package (Version 9, Omicsoft Corporation, Research Triangle Park, NC, United States). The levels of gene expression were obtained by subtracting first from the individual Cq values a geometric average normalizing value of four reference genes, namely B2M, Eif2b1, Gusb, and Ywhaz. Relative expression was then calculated as potency of this difference in Cq with the basis of 2.

### Statistics

All data are presented as mean ± SEM (standard error of the mean). The n-value given in the figures correspond to the number of animals tested. Differences between concentration-response relationships were tested by ANOVA for repeated measurements. The confidence level *p* was set to 0.05.

## Results

### Vascular Activities of Structurally Different ET-CORMs

**FIGURE 1:**
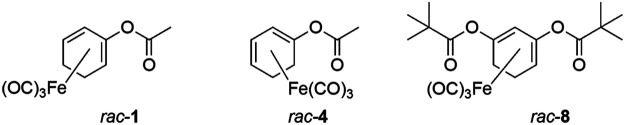
Chemical structure of the ET-CORMs *rac*-**1**, *rac-* **4**, and *rac*-**8**.

**FIGURE 2:**
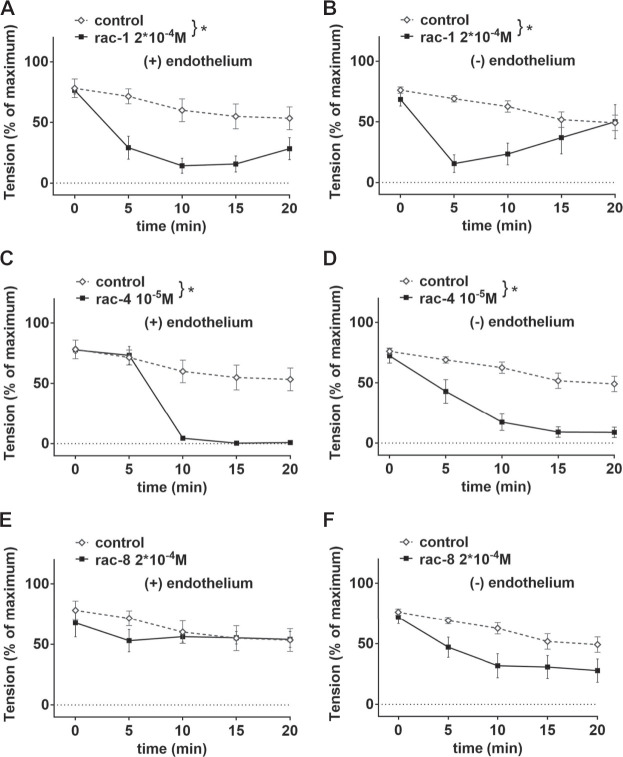
Vascular activities of structurally different ET-CORMs on mesenteric arteries. **(A)** Effect of *rac-* **1** (*rac*-**1** 2*10^–4^ M) and of DMSO (control) on vessel tension induced by 10^–6^ M methoxamine in endothelium-intact arteries. (repeated measures ANOVA, n = 5, *p* < 0.05). **(B)** Effect of *rac-* **1** (*rac-* **1** 2*10^–4^ M) and of DMSO (control) on vessel tension induced by 10^–6^ M methoxamine in endothelium-denuded arteries. (repeated measures ANOVA, n = 5, *p* < 0.05). **(C)** Effect of *rac-* **4** (*rac-* **4** 10^–5^ M) and of DMSO (control) on vessel tension induced by 10^–6^ M methoxamine in endothelium-intact arteries. (repeated measures ANOVA, n = 5, *p* < 0.05). **(D)** Effect of *rac-* **4** (*rac-* **4** 10^–5^ M) and of DMSO (control) on vessel tension induced by 10^–6^ M methoxamine in endothelium-denuded arteries. (repeated measures ANOVA, n = 5, *p* < 0.05). **(E)** Effect of *rac-* **8** (*rac-* **8** 2*10^–4^ M) and of DMSO (control) on vessel tension induced by 10^–6^ M methoxamine in endothelium-intact arteries. (repeated measures ANOVA, n = 5, *p* = 0.60). **(F)** Effect of *rac*-**8** (*rac-* **8** 2*10^–4^ M) and of DMSO (control) on vessel tension induced by 10^–6^ M methoxamine in endothelium-denuded arteries. (repeated measures ANOVA, n = 5, *p* = 0.06).

Since the positions of the ester functionality in ET-CORMs, as well as the mother compound from which they are derived, strongly influence their biological activity (Romanski et al., 2013), we first tested three structural different ET-CORMs (Figure 1) for vascular activity. The tested concentrations were chosen on the basis of preliminary experiments assessing the toxicity of these ET-CORMs. Pre-contracted mesenteric arteries strongly dilated upon treatment with both types of acetate containing ET-CORMs (*rac-* **1**, Figures 2A,B; *rac-* **4**, Figures 2C,D), while treatment with the pivalate containing ET-CORM (*rac*-**8**) resulted in no vasodilation (Figures 2E,F). For the former types of ET-CORMs vasodilation occurred irrespective of the presence or absence of the endothelium (Figures 2A–D). In order to focus on smooth muscle cell signaling all further experiments have been performed on endothelium-denuded vessels. Vasodilation appeared to be transient for *rac-* **1**, particularly in denuded arteries, whereas for *rac-* **4** arteries remained dilated over the whole observation time.

### ET-CORM Mediated Vascular Activity Is Guanylyl Cyclase Dependent

**FIGURE 3:**
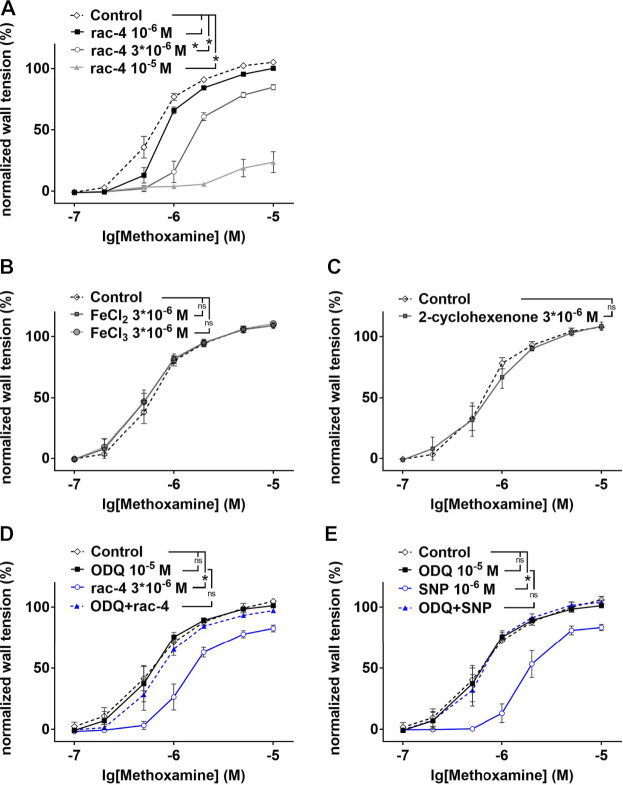
Dilation of mesenteric arteries induced by *rac-* **4** is guanylyl cyclase dependent. **(A)** Methoxamin-induced contraction in the absence of any substances (Control) and in the presence of different concentrations of *rac-* **4** (10^–6^ M, 3*10^–6^ M, 10^–5^ M). (repeated measures ANOVA, n = 6, *p* < 0.05, particular comparisons indicated in legend). **(B)** Methoxamin-induced contraction in the absence of any substances (Control) and in the presence of 3*10^–6^ M FeCl_2_ and FeCl_3_, respectively. (repeated measures ANOVA, n = 6, *p* = 0.68 and *p* = 0.53 for FeCl_2_ and FeCl_3_, respectively). **(C)** Methoxamin-induced contraction in the absence of any substances (Control) and in the presence of 3*10^–6^ M 2-cyclohexenone. (repeated measures ANOVA, n = 5, *p* = 0.77). **(D)** Methoxamin-induced contraction in the absence of any substances (Control), in the presence of 10^–5^ M ODQ, in the presence of 3*10^–6^ M rac-4 and in the presence of the combination of ODQ and *rac-* **4**. (repeated measures ANOVA, n = 6, Control vs *rac-* **4**: *p* < 0.05; Control vs ODQ: *p* = 0.79; ODQ vs ODQ + rac-4: *p* = 0.26). **(E)** Methoxamin-induced contraction in the absence of any substances (Control), in the presence of 10^–5^ M ODQ, in the presence of 10^–6^ M SNP and in the presence of the combination of ODQ and SNP. (repeated measures ANOVA, n = 6, Control vs SNP: *p* < 0.05; Control vs ODQ: *p* = 0.77; ODQ vs ODQ + SNP: *p* = 0.90).

In as much as *rac-* **4** was most effective in inducing vasodilation at a relative low concentration compared to the other ET-CORMs tested, subsequent experiments to delineate the underlying mechanisms were only performed with *rac*-**4**. As shown in Figure 3A, vasodilation by *rac-* **4** occurred in a concentration-dependent manner. To exclude that vasodilation was mediated *via* the byproducts of *rac-* **4** hydrolysis rather than CO, FeCl_2_, FeCl_3_ and 2-cyclohexanone were applied to the vessels. None of these compounds lead to vasodilation (Figures 3B,C), suggesting that CO released from *rac-* **4** is likely responsible for modulating vascular tone. Pre-treatment of mesenteric arteries with the guanylyl cyclase inhibitor ODQ abolished *rac-* **4** mediated vasodilation (Figure 3D), similar as for the known guanylyl cyclase activator SNP (Figure 3E).

### ET-CORM Mediated Vascular Activity Is Mediated by Potassium Channels

**FIGURE 4:**
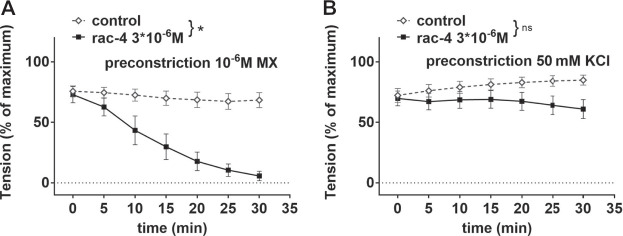
Dilation of mesenteric arteries induced by rac-**4** is K-channel dependent. **(A)** Effect of *rac*-**4** (rac-4 3*10^–6^ M) and of DMSO (control) on vessel tension induced by 10^–6^ M methoxamine. (repeated measures ANOVA, n = 6, *p* < 0.05). **(B)** Effect of *rac-* **4** (rac-4 3*10^–6^ M) and of DMSO (control) on vessel tension induced by 50 mM KCl. (repeated measures ANOVA, n = 8, *p* = 0.14).

In order to address whether *rac-* **4**-induced vasodilation is mediated by potassium channels, mesenteric arteries were pre-treated with 50 mM KCl. Under these conditions, the efflux of K^+^ through potassium channels in smooth muscle cells is abolished due to a lack of driving force for the ions, thereby eliminating the functionality of potassium channels. *Rac-* **4** produced considerable relaxation of methoxamine pre-contracted mesenteric arteries (Figure 4A). An effect of *rac-* **4** on KCl pre-contracted arteries was not detected (Figure 4B), indicating an involvement of potassium channels in the vasodilatory action of *rac-* **4**.

**FIGURE 5:**
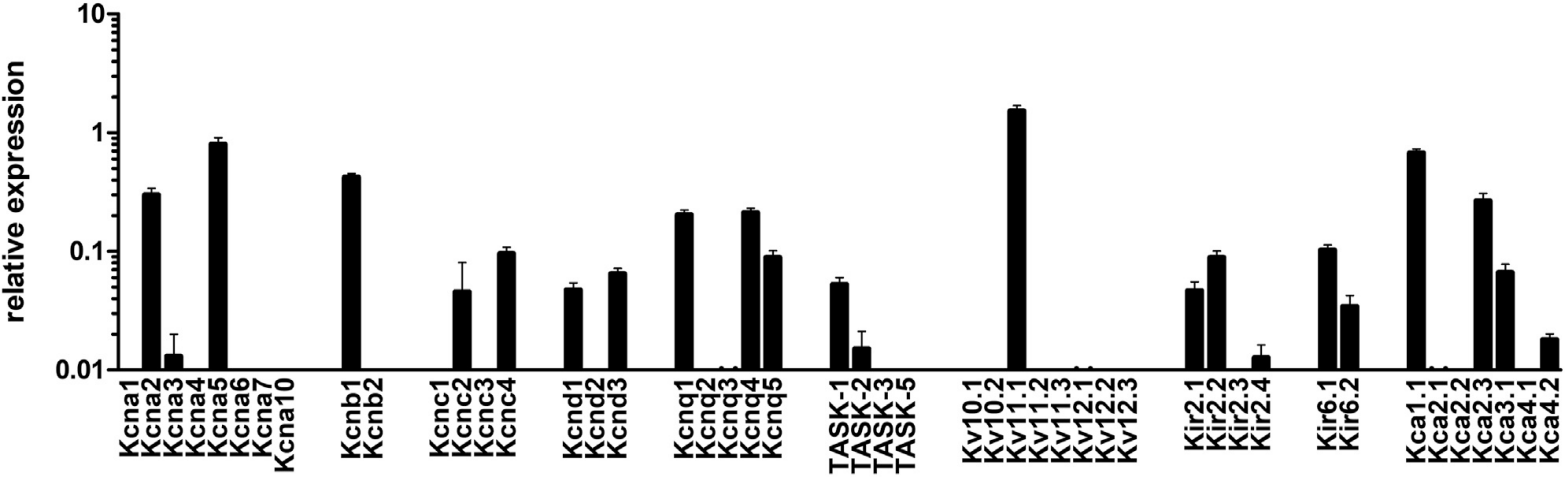
mRNA expression of K-channels in mesenteric arteries. Relative expression of K (+)-channels assessed by realtime PCR with Taqman primers in intact mesenteric arteries. Normalized was performed as described in the method sections relative to the expression of five reference (housekeeping) genes, B_2_M, Eif2b1, Gusb, Myh11, and Ywhaz (n = 8).

In subsequent experiments we sought to assess which potassium channels were mediating *rac*-**4**-induced vasodilation. To this end, we first determined gene expression levels of various potassium channels in mesenteric arteries by qPCR. As shown in Figure 5, Kv1 (KCNA), Kv2 (KCNB), Kv3 (KCNC), Kv7 (KCNQ), Kv11, Kir2, Kir6, BK (Kca1) and SK (Kca2) channels are abundantly expressed in these arteries. Of note, Kv3, Kv11, Kir2 and SK channels were not further explored because of a lack of specific inhibitors or a lack of published data suggesting their involvement in guanylyl cyclase-mediated vasodilation. The contribution of the other potassium channels to *rac-* **4** mediated vasodilation was studied using specific channel inhibitors as depicted in Table 1.

**TABLE 1:** combination of potassium channel inhibitors used for leaving only a particular channel available.

Nr.	Combination of potassium channel inhibitors^a^	Available channel	Blocked channel
1	Iberiotoxin, XE991, DPO-1, STTX, Glibenclamid	Kir2	BK, Kv7, Kv1, Kv2, Kir6
2	Iberiotoxin, XE991, DPO-1, STTX, BaCl_2_	Kir6	BK, Kv7, Kv1, Kv2, Kir2
3	XE991, DPO-1, STTX, Glibenclamid, BaCl_2_	BK	Kv7, Kv1, Kv2, Kir6, Kir2
4	Iberiotoxin, Glibenclamid, BaCl_2_	Kv	BK, Kir6, Kir2

a Concentration used: Iberiotoxin (BK) 0.1 µM (Galvez et al., 1990); XE991 (Kv7) 3 µM (Greenwood and Ohya, 2009; Zavaritskaya et al., 2020); DPO-1 (Kv1) 1 µM (Lagrutta et al., 2006; Tsvetkov et al., 2016); Stromatoxin (STTX) (Kv2) 0.1 µM (Escoubas et al., 2002); BaCl_2_ (Kir2) 30 µM (Schubert et al., 2004); Glibenclamide (Kir6) 1 µM (Schubert et al., 1997).

**FIGURE 6:**
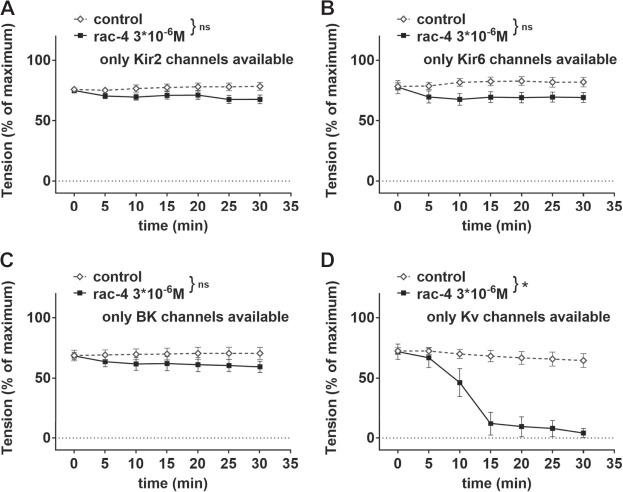
Dilation of mesenteric arteries induced by *rac*-**4** is Kv-channel dependent. **(A)** Effect of *rac-* **4** (*rac-* **4** 3*10^–6^ M) and of DMSO (control) on vessel tension induced by methoxamine together with a mixture of K-channel blockers (10^−7^ M Iberiotoxin, 3*10^−6^ M XE991, 10^–6^ M DPO-1, 10^−7^ M stromatoxin, 10^−6^ M glibenclamide) leaving only Kir2 channels available. Of note, both treatments reached the same level of vessel tension. (repeated measures ANOVA: n = 8; *p* = 0.09). **(B)** Effect of *rac*-**4** (*rac*-**4** 3*10^–6^ M) and of DMSO (control) on vessel tension induced by methoxamine together with a mixture of K-channel blockers (10^−7^ M Iberiotoxin, 3*10^−6^ M XE991, 10^–6^ M DPO-1, 10^−7^ M stromatoxin, 3*10^−5^ M BaCl_2_) leaving only Kir6 channels available. Of note, both treatments reached the same level of vessel tension. (repeated measures ANOVA: n = 8; *p* = 0.07). **(C)** Effect of *rac-* **4** (*rac-* **4** 3*10^–6^ M) and of DMSO (control) on vessel tension induced by methoxamine together with a mixture of K-channel blockers (3*10^−6^ M XE991, 10^–6^ M DPO-1, 10^−7^ M stromatoxin, 3*10^−5^ M BaCl_2_, 10^−6^ M glibenclamide) leaving only BK channels available. Of note, both treatments reached the same level of vessel tension. (repeated measures ANOVA: n = 7; *p* = 0.28). **(D)** Effect of *rac-* **4** (*rac*-**4** 3*10^–6^ M) and of DMSO (control) on vessel tension induced by methoxamine together with a mixture of K-channel blockers (10^−7^ M Iberiotoxin, 3*10^−5^ M BaCl_2_, 10^−6^ M glibenclamide) leaving only Kv channels available. Of note, both treatments reached the same level of vessel tension. (repeated measures ANOVA: n = 7; *p* < 0.05).

Further, mesenteric arteries were pre-treated with different combinations of potassium channel inhibitors leaving only Kir2, Kir6, BK or Kv channels, respectively available (Table 1). When compared to the effect of its solvent DMSO, *rac-* **4** induced only vasodilation when Kv channels were not inhibited (Figures 6A–D).

**FIGURE 7:**
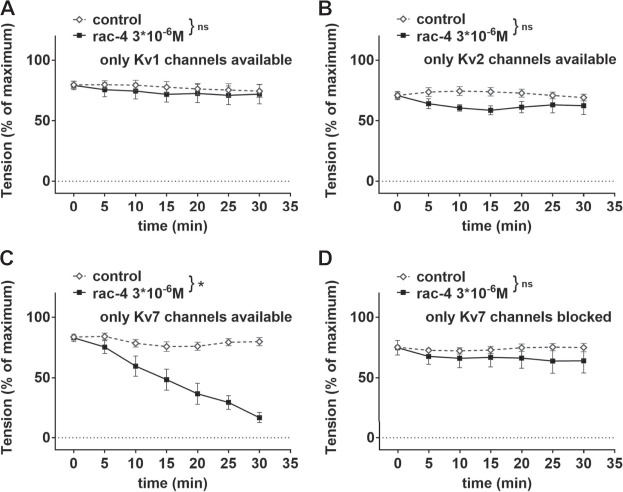
Dilation of mesenteric arteries induced by *rac-* **4** is Kv7-channel dependent. **(A)** Effect of *rac*-**4** (*rac-* **4** 3*10^–6^ M) and of DMSO (control) on vessel tension induced by methoxamine together with a mixture of K-channel blockers (10^−7^ M Iberiotoxin, 3*10^−6^ M XE991, 10^−7^ M stromatoxin, 3*10^−5^ M BaCl_2_, 10^−6^ M glibenclamide) leaving only Kv1 channels available. Of note, both treatments reached the same level of vessel tension. (repeated measures ANOVA: n = 6; *p* = 0.63). **(B)** Effect of *rac*-**4** (*rac-* **4** 3*10^–6^ M) and of DMSO (control) on vessel tension induced by methoxamine together with a mixture of K-channel blockers (10^−7^ M Iberiotoxin, 3*10^−6^ M XE991, 10^–6^ M DPO-1, 3*10^−5^ M BaCl_2_, 10^−6^ M glibenclamide) leaving only Kv2 channels available. Of note, both treatments reached the same level of vessel tension. (repeated measures ANOVA: n = 7; *p* = 0.09). **(C)** Effect of *rac-* **4** (*rac*-**4** 3*10^–6^ M) and of DMSO (control) on vessel tension induced by methoxamine together with a mixture of K-channel (10^−7^ M Iberiotoxin, 10^–6^ M DPO-1, 10^−7^ M stromatoxin, 3*10^−5^ M BaCl_2_, 10^−6^ M glibenclamide) leaving only Kv7 channels available. Of note, both treatments reached the same level of vessel tension. (repeated measures ANOVA: n = 10; *p* < 0.05). **(D)** Effect of *rac-* **4** (*rac*-**4** 3*10^–6^ M) and of DMSO (control) on vessel tension induced by methoxamine together with 3*10^−6^ M XE991 blocking only Kv7 channels. Of note, both treatments reached the same level of vessel tension. (repeated measures ANOVA: n = 6; *p* = 0.42).

In order to identify the Kv channel subtype involved, mesenteric arteries were pre-treated with different combinations of potassium channel inhibitors leaving only Kv1, Kv2 or Kv7 channels available (Table 2). *Rac-* **4** did not induce vasodilation compared to the effects of its solvent DMSO when only Kv1 (Figure 7A) or only Kv2 (Figure 7B) channels were available. In contrast, when only Kv7 channels were left available *rac-* **4** was able to dilate the vessels (Figure 7C). In line with this, inhibition of Kv7 channels only by XE991 completely abolished *rac-* **4** induced vasodilation (Figure 7D).

**TABLE 2:** Combination of Kv channel inhibitors used for leaving only a particular channel available.

Nr.	Combination of potassium channel inhibitors^a^	Available channel	Blocked channel
1	Iberiotoxin, XE991, STTX, Glibenclamid, BaCl_2_	Kv1	BK, Kv7, Kv2, Kir6, Kir2
2	Iberiotoxin, XE991, DPO-1, Glibenclamid, BaCl_2_	Kv2	BK, Kv7, Kv1, Kir6, Kir2
3	Iberiotoxin, DPO-1, STTX, Glibenclamid, BaCl_2_	Kv7	BK, Kv1, Kv2, Kir6, Kir2
4	XE991	BK, Kv1, Kv2, Kir6, Kir2	Kv7

a Concentration used: Iberiotoxin (BK) 0.1 µM (Galvez et al., 1990); XE991 (Kv7) 3 µM (Greenwood and Ohya, 2009; Zavaritskaya et al., 2020); DPO-1 (Kv1) 1 µM (Lagrutta et al., 2006; Tsvetkov et al., 2016); Stromatoxin (STTX) (Kv2) 0.1 µM (Escoubas et al., 2002); BaCl_2_ (Kir2) 30 µM (Schubert et al., 2004); Glibenclamide (Kir6) 1 µM (Schubert et al., 1997).

## Discussion

Carbon monoxide (CO) is an autocrine and paracrine vasodilator that can regulate tone in resistance vessels throughout the body (Leffler et al., 1999). While it has been suggested that gaseous CO mediates vasodilation through the activation of large-conductance Ca^2+^-activated potassium (BK_Ca_) channels as well as sGC present in arterial smooth muscle cells (Wang et al., 1997b; Jaggar et al., 2005), the role of the former has been questioned when applying the CO-donor CORM-2 (Wang et al., 1997b) but not CORM-A1 (Ryan et al., 2006) on femoral or renal interlobar arteries respectively. Whether this is due to differential activation of K^+^-channels by different CORMs or due to differences in the expression level of K^+^-channels in different vessels is still elusive. The cytoprotective -, anti-inflammatory - and CO releasing properties of ET-CORMs have been extensively studied (Ryan et al., 2006; Romanski et al., 2013; Stamellou et al., 2014), yet if ET-CORMs also are vasoactive on resistance vessels remains to be addressed. Based on the current understanding of the vasoactive properties of CO and CORMs, this study underlies the hypothesis that ET-CORMs are able to cause vasodilation of pre-contracted resistance vessels in a sGC and K^+^-channel dependent manner. The main findings of this study are as follows. 1) Pre-contracted mesenteric arteries strongly dilate upon treatment with both types of acetate containing ET-CORMs (*rac*-**1** and *rac*-**4**), while treatment with the pivalate containing ET-CORM (*rac*-**8**) resulted in no vasodilation. 2) Pre-treatment of mesenteric arteries with the sGC inhibitor ODQ abolished *rac*-**4** mediated vasodilation, similar as for the known sGC activator SNP. 3) A variety of K^+^-channels are expressed in mesenteric arteries. The relative expression of each largely differs. 4) *rac*-**4** mediated vasodilation occurs when only Kv7 channels are available and is blocked by the Kv7 inhibitor XE991. This suggests that Kv7 channels are the main potassium channels by which *rac*-**4** mediates vasodilation in the vessels studied.

In previous studies, we revealed that the anti-inflammatory and cytoprotective properties of ET-CORMs strongly depend on their chemical structure, more specifically on the mother compound they are derived from and the type and position of the ester functionality that they harbor (Stamellou et al., 2014). In the present study, we show that this also holds true for the vasorelaxation properties of ET-CORMs. The efficacy of the acetate containing ET-CORMs, *rac*-**1** and *rac*-**4** was similar as reported for the inhibition of VCAM-1 (Romanski et al., 2013; Stamellou et al., 2014), with *rac*-**4** being more effective compared to *rac*-**1**. This correlates with the slower release of CO from *rac*-**1** (Romanski et al., 2012b). In contrast to the acetate containing ET-CORMs, *rac*-**8** was not able to cause vasodilation of pre-contracted mesenteric arteries, an unexpected finding based on previous published biological properties that were similar for *rac*-**1** and *rac*-**8**. However, it should be emphasized that previous experiments with *rac*-**8** were performed in human cells, which, compared to rat mesenteric arteries, may express different types of esterase enzymes for hydrolysis of the pivalate ester functionality. Moreover, uptake and hydrolysis of *rac*-**8** by mesenteric arteries may require more time than the 30-min time frame in which vasodilation was recorded. Previous publications have indeed demonstrated differences in hydrolysis efficiencies of acetate and pivalate propanolol prodrugs (Takahashi et al., 1995) and acyloxy nitroso compounds (Shoman et al., 2011) Likewise, the finding that pig liver esterase-induced CO release from pivalate containing ET-CORMs is much slower compared to that of the corresponding acetate containing ET-CORMs (Romanski et al., 2012b) corroborates the current results obtained with *rac*-**1**, *rac*-**4** and *rac*-**8**. It should also be noted that the cytoprotective properties of ET-CORMs we reported previously were attributed to the inhibition of the NFκB–and activation of the Nrf2-Keap1 pathways (Ryan et al., 2006). These pathways are not involved in vasorelaxation.

Our study demonstrates that *rac*-**4** mediates vasorelaxation in mesenteric arteries irrespective of a disrupted endothelium. In contrast, Alshehri et al. (Alshehri et al., 2013) reported that CORM-3 mediated vasorelaxation is partly endothelium dependent in the largest conduit vessel of the organism, the aorta. Differences in the vessel studied, concentrations used (100 µM vs. in 10 μM, CORM-3 vs. *rac*-**4**) and intrinsic properties of the CORMs may underlie this discrepancy. In line with our current findings, most studies, including the study of Alshehri et al. (Alshehri et al., 2013), report that CO and CORM mediated vasorelaxation involves activation of the sGC (De Backer and Lefebvre, 2007; van der Sterren et al., 2011; Magierowska et al., 2019), even though sGC binds CO only with limited affinity (Martin et al., 2006). There are also studies however in which sGC did not participate in CO mediated vasorelaxation. As such ODQ did not inhibit, but rather potentiated endothelium independent relaxation by CORM-3 in WKY rat aorta rings (Achouh et al., 2005). Also, in the study of Decaluwé K et al. (Wang et al., 1997b) it was reported that vasorelaxation by gaseous CO strongly depends on sGC activation, while ODQ had no effect on CORM-2 induced relaxations of mice femoral arteries.

The proficiency of CO to regulate a variety of ion channels, e.g., calcium-activated K^+^ (BK_Ca_) channels (Jaggar et al., 2005), voltage-activated K^+^ (Kv) channels (Dallas et al., 2011), and Ca^2+^ channels (Duckles et al., 2015), has been demonstrated in many studies and reflects the pluripotency of CO to participate in numerous physiological processes and pathologies. Likewise, the involvement of K^+^-channels in CO mediated vasorelaxation is unequivocally reported, albeit that there is ambiguity with respect to the class of K^+^-channels that are supposedly involved. While a number of studies have suggested an association between HO and BK_Ca_ channels (Williams et al., 2004; Kooli et al., 2008) or their activation by CO (Wang et al., 1997a; Jaggar et al., 2002; Yi et al., 2010), other studies have postulated a role for voltage-gated K^+^-channels (Kv1.5, Kv11.1) (Dong et al., 2007). Our data on *rac*-**4** mediated vasorelaxation do not support the involvement of BK_Ca_ channels, but instead we observed that *rac*-**4** induced only significant vasodilation when Kv7 channels were not inhibited. Failure of *rac*-**4** to activate BK_Ca_ channels is unlikely due to differences in the relative expression between BK_Ca_ (Kca1) and Kv7 (KCNQ) as the mRNA expression of the former was even slightly higher. The mechanism by which CO affects BK_Ca_ is ambiguously discussed (Wang et al., 1997b; Jaggar et al., 2005) (De Backer and Lefebvre, 2007). In the studies of Jaggar et al. (Jaggar et al., 2005) CO activates BK_Ca_ channels by binding to channel-bound heme, which alters the interaction between heme and the conserved heme-binding domain of the channel. Their data corroborate previous findings of Wang et al. (Wang et al., 1997b) who showed that the effect of gaseous CO on K_Ca_ channels was not mediated by cGMP, but contradict the findings of De Backer et al. (De Backer and Lefebvre, 2007) in which CO mediated relaxation in circular smooth muscle strips of the murine gastric fundus and jejunum was sGC and BK_Ca_ channel dependent. Due to different releasing mechanisms described for CORMs it is conceivable that steady-state intracellular CO concentrations will be reached faster by gaseous CO than by CORMs. This is particularly the case for ET-CORMs, which require further intra-cellular processing before CO is released (Romanski et al., 2012a). Hence, higher CO concentrations may favor the interaction with heme in BK_Ca_, while the relatively small amount of CO released for rac-4 within the timeframe of the experiment may not suffice to activate BK_Ca_ channels. We postulate that *rac*-**4** stimulates sGC, resulting in the activation of cGMP-dependent protein kinase (PKG) that subsequently phosphorylates and activates Kv7 channels. With the exception of Kv7.1 a conserved PKG phosphorylation site, i.e., KFKE (T/S)LRPY, is present in all other Kv7 members. PKG mediated Threonine or Serine phosphorylation of this site was predicted by the NetPhos 3.1 server. Assessment of cGMP and phosphorylation of Kv7 in mesenteric arteries is warranted to strengthen this assumption.

Ruthenium-containing carbonyl complexes such as tricabonyldichlororuthenium (II) dimer (CORM-2) or [Ru(CO)3Cl (glycinate)] (CORM-3) can activate K^+^-channels directly and independent of CO release (Dong et al., 2007; Hou et al., 2008; Williams et al., 2008; Riddle and Walker, 2012; Gessner et al., 2017) Hydrolysis of *rac*-**4** results in the generation of CO, acetic acid, 2-cyclohexanone and Fe^2+/3+^ in a molar ratio of 3:1 (CO: break-down product). Although our study does not provide formal proof that *rac*-**4** mediated vasorelaxation exclusively involves CO, none of the break-down products were able to mediate vasodilation of pre-contracted mesenteric arteries. It should be emphasized however, that hydrolysis of *rac*-**4** occurs intra-cellular, yet it is not clear to what extent each of the break-down products are taken up by the mesenteric arteries when applied in the myograph.

In conclusion our current study has clearly demonstrated that the acetate containing ET-CORMs can mediate vasorelaxation of small mesenteric arteries in a sGC and Kv7 dependent manner. Both in rodents and human Kv7 proteins are expressed in the smooth muscle layer of visceral arteries (Ng et al., 2011; Haick and Byron, 2016), where they regulate contractility and vascular resistance. If all Kv7 subtypes are activated by *rac*-**4** remains to be addressed. While our study uses an *ex-vivo* model for assessing vascular responses, *in vivo* regulation of vascular tone is more complex. Yet, in keeping with the potential role of Kv7 channels in cardiovascular disease (Brenyo et al., 2012), hypertension (Carr et al., 2016), obesity and diabetes (Yamagata et al., 2011), these promising *ex-vivo* data warrant further *in vivo* studies in relevant disease models to assess the therapeutic potential of ET-CORMs.

## Data Availability

The raw data supporting the conclusions of this article will be made available by the authors, without undue reservation.
